# Determinants of post-COVID-19 symptoms among adults aged 55 or above with chronic conditions in primary care: data from a prospective cohort in Hong Kong

**DOI:** 10.3389/fpubh.2023.1138147

**Published:** 2023-05-05

**Authors:** Dexing Zhang, Vincent Chi-Ho Chung, Dicken Cheong-Chun Chan, Zijun Xu, Weiju Zhou, King Wa Tam, Rym Chung-Man Lee, Regina Wing-Shan Sit, Stewart W. Mercer, Samuel Yeung-Shan Wong

**Affiliations:** ^1^JC School of Public Health and Primary Care, The Chinese University of Hong Kong, Shatin, Hong Kong SAR, China; ^2^Usher Institute, University of Edinburgh, Edinburgh, United Kingdom

**Keywords:** COVID-19, primary care, older adults, infection, post-acute and long COVID-19 symptoms, predictors

## Abstract

**Background:**

Primary care patients, especially those with an older age, are one of the most vulnerable populations for post-COVID-19 symptoms. Identifying predictors of post-COVID symptoms can help identify high-risk individuals for preventive care.

**Methods:**

Out of 977 primary care patients aged 55 years or above with comorbid physical and psychosocial conditions in a prospective cohort in Hong Kong, 207 patients infected in the previous 5–24 weeks were included. The three most common post-COVID-19 symptoms (breathlessness, fatigue, cognitive difficulty), which lasted beyond the 4-week acute infection period, were assessed using items from the COVID-19 Yorkshire Rehabilitation Scale (C19-YRS), together with other self-reported symptoms. Multivariable analyses were conducted to identify predictors of post-acute and long COVID-19 symptoms (5–24 weeks after infection).

**Results:**

The 207 participants had a mean age of 70.8 ± 5.7 years, 76.3% were female, and 78.7% had ≥2 chronic conditions. In total, 81.2% reported at least one post-COVID symptom (mean: 1.9 ± 1.3); 60.9, 56.5 and 30.0% reported fatigue, cognitive difficulty, and breathlessness respectively; 46.1% reported at least one other new symptom (such as other respiratory-related symptoms (14.0%), insomnia or poor sleep quality (14.0%), and ear/nose/throat symptoms (e.g., sore throat) (10.1%), etc.). Depression predicted post-COVID-19 fatigue. The female sex predicted cognitive difficulty. Receiving fewer vaccine doses (2 doses vs. 3 doses) was associated with breathlessness. Anxiety predicted a higher overall symptom severity level of the three common symptoms.

**Conclusion:**

Depression, the female sex, and fewer vaccine doses predicted post-COVID symptoms. Promoting vaccination and providing intervention to those at high-risk for post-COVID symptoms are warranted.

## 1. Introduction

By 28 February 2023, over 758 million confirmed cases of COVID-19 and 6.86 million deaths were reported to the WHO (1). It is foreseen that many more people worldwide would get COVID-19 eventually. After the acute period in the initial few weeks, many people were found to have post-acute COVID-19 (5–12 weeks after infection) symptoms and long-term COVID-19 (more than 12 weeks after infection) symptoms (2). Fatigue, cognitive problems, breathlessness, headache, coughing, chest pain, hair loss, decreased mental status, and olfactory dysfunction are frequently persistent symptoms (3–7). The pandemic has placed a heavy burden on rehabilitation care for COVID-19 survivors around the world. Systematic reviews of different age groups and infection severities to date have consistently found a high prevalence of post-COVID symptoms (mean follow-up period from 3 weeks to 7 months) (3–6). Virus variants of concern, which include alpha, beta, delta and now omicron, have suggested that COVID-19 might become endemic and would continue its enormous impacts, especially on vulnerable populations including older adults, who usually have multiple chronic conditions and face higher risks for infection and post-COVID symptoms (8).

Understanding patients’ post-COVID symptoms status and relevant predictors is important for providing better preventive and rehabilitation services. However, it is yet unclear and needs more epidemiological studies with low recall bias to understand predictors of post-COVID symptoms among high-risk populations (9, 10). Currently, only a few longitudinal cohort studies have examined predictors of post-COVID symptoms in primary care (11–14) and further prospective studies are needed to determine the risk factors predicting post-COVID symptoms, especially among older patients in primary care who are more vulnerable (8). Based on a prospective cohort study that examines the physical and psychosocial needs of older patients with comorbid physical and psychosocial conditions in primary care, this study explored various predictors of the three most common post-COVID symptoms (fatigue, cognitive problems, and breathlessness) (3–7).

## 2. Materials and methods

### 2.1. Study design and setting

This study was based on a prospective cohort study (unpublished yet) among 1,440 older patients in public primary care clinics in Hong Kong, where the number of COVID-19 cases remained low (about 12,000 cases) until the 5th wave of Omicron dominant outbreak starting in late December 2021 (15, 16). The number of infected cases reached as high as 56,827 in a single day in early March 2022, and the government reporting system recorded 749,318 and 450,146 cases confirmed by nucleic acid amplification tests (NAATs) or rapid antigen tests (RATs), respectively, in the total population of 7.6 million (15.8%) as of May 2022 (15–17).

### 2.2. Participants

Participants were from an existing cohort. The original aim of the cohort was to identify the unique health needs of older primary care patients with both physical and psychosocial issues such that corresponding complex interventions can be developed to address their needs. The inclusion criteria were (1) Chinese; (2) aged 55 and above; (3) with at least one physical condition (e.g., hypertension, diabetes mellitus, chronic pain, sarcopenia) plus at least one mental/social condition (e.g., depression, anxiety, mild cognitive impairment, loneliness) (Supplementary Table S1). The exclusion criteria were (1) psychosis or bipolar disorder; (2) being actively suicidal; (3) receiving services for substance abuse; (4) receiving psychological therapy from a psychologist within the past 6 months.

### 2.3. Data collection

Pre- and post-5th wave outbreak assessments were conducted from November 2019 to May 2021 (pre-5th wave) and from April to May 2022 (during the 5th wave), respectively. Trained nurses, social workers, and research assistants conducted face-to-face pre-assessments in a public primary care outpatient clinic affiliated with an academic unit. The post-assessments were conducted over the telephone, with at least 3 phone calls made at different times on different days for unanswered calls. A preset database in REDCap (Research Electronic Data Capture) was used for baseline assessment and an online questionnaire was used simultaneously during the post-assessments. Score ranges and logic settings were set up in both databases to ensure the data entry quality.

### 2.4. Measures

Measures include basic socio-demographics (age, sex, and Comprehensive Social Security Assistance (CSSA) Scheme reception for low-income families), body mass index (BMI), waist, alcohol drinking behaviour, the number of chronic conditions, physical activity level, pain, sarcopenia, frailty, cognitive function, depression, anxiety, social support, and loneliness. All the scales have been validated (details below) and widely used, including in our previous study in Hong Kong (18). Except for COVID-19 vaccination information, all the independent variables below (2.4.1–2.4.10) were collected at baseline. Post-COVID-19 symptoms (dependent variable) were collected at follow-up.

#### 2.4.1. Number of chronic conditions

The number of chronic diseases was collected via self-report and information retrieved from the public Clinical Management System (CMS) (18). The chronic condition list contained 43 chronic conditions with an additional question on other diseases (Supplementary Table S2). This was based on the International Statistical Classification of Diseases 11 and used in previous local studies (19, 20).

#### 2.4.2. Depression and anxiety

The 9-item Patient Health Questionnaire (PHQ-9) (21) and the 7-item Generalised Anxiety Disorder (GAD-7) (22) scales were used to measure depression and anxiety, respectively. Both scales have been validated with acceptable psychometric properties among the Chinese population.

#### 2.4.3. Loneliness and perceived social support

Loneliness was measured by the validated 6-item De Jong Gierveld Loneliness Scale (DJGLS) (23). The DJGLS has a total loneliness score besides two subscales on social and emotional loneliness. The perceived social support was measured by the validated Multidimensional Scale of Perceived Social Support (MSPSS) (24). Higher scores represent higher loneliness/social support levels.

#### 2.4.4. Physical activity level

Physical activity level was measured by the validated Chinese version of the Physical Activity Scale for the Elderly (PASE-C) (25). Higher scores denote being more active.

#### 2.4.5. Alcohol drinking

The validated 3-item Alcohol Use Disorders Identification Test-consumption (AUDIT-C) was used (26). It has satisfactory accuracy (0.83), a high negative predictive value (0.93) and a moderate level of positive predictive value (0.64) with the cut-off at ≥5 (26).

#### 2.4.6. Sarcopenia

Sarcopenia was measured by a simple five-item Sarcopenia Assessment (SARC-F). It has been validated among Chinese and shown to have excellent specificity for screening sarcopenia with the cut-off at ≥4 (27, 28).

#### 2.4.7. Frailty

Frailty was measured by the validated FRAIL scale. It has five items, each with a yes (1) or no (0) answer (score range: 0–5). A score of 1–2 denotes pre-frailty, and a score of 3–5 denotes frailty (29).

#### 2.4.8. Pain

The pain severity score was measured by the subscale of the validated Chinese version of the Brief Pain Inventory (BPI) (30). It rated worst pain, least pain, average pain, and pain right now in the past week, on a scale of 0 (no pain)–10 (the worst pain one can imagine). Higher scores mean higher severity.

#### 2.4.9. Cognitive function

The validated Hong Kong Montreal Cognitive Assessment (HK-MoCA) was used (score range: 0–30). A lower score suggests poorer cognitive function, adjusting for years of education (+1 point if <6 years of education) (31). The staff who conducted the assessment had the certification for using HK-MoCA.

#### 2.4.10. COVID-19 vaccination

COVID-19 vaccine type and dose information was collected at follow-up.

#### 2.4.11. Post-COVID-19 symptoms (dependent variable)

During follow-up, COVID-19 infection status was asked over the telephone for their results of either compulsory tests or self-tests (either using NAATs or RATs). Post-COVID-19 symptoms were symptoms that persisted beyond the initial acute infection period. The period defining post-acute and long COVID-19 symptoms was 5–24 weeks after infection: (2) post-acute COVID symptoms (week 5 to week 12) and long COVID symptoms (week 12 to week 24). Three most common post-COVID symptoms (breathlessness, fatigue and cognitive difficulty) (3–7) were asked using items from the self-reported COVID-19 Yorkshire Rehabilitation Scale (C19-YRS). The C19-YRS is an outcome measure for long COVID symptoms with high internal consistency (Cronbach’s α = 0.891) (32). The National Institute for Health and Clinical Excellence (NICE) guideline has advocated its use in all long-term COVID clinics in the UK. The scale has also been translated into multiple languages. It has been translated and back-translated into Chinese by bi-linguists. Each item was rated on an 11-point scale from 0 (none of this symptom) to 10 (extremely severe level or impact). Overall, the severity score with a range of 0 to 10 was the mean of the available symptom severity scores from the three post-COVID symptoms. In addition, an open-ended question was asked to understand if any other new symptoms had emerged since the infection, with a severity score (range: 0–10).

### 2.5. Statistical methods

Both univariable analysis (t-tests or chi-square tests) and multivariable logistic regression were conducted. The dependent variable was post-COVID symptoms, which were analyzed as categorical variables using two different methods: (1) having any of the three post-acute symptoms, respectively; (2) an overall symptom severity score, with the score collapsed into two groups (1–5 mild problem; 6–10 moderate or severe problem). The grouping was identified as recommended by the modified C19-YRS scale (33). *Post hoc* rescoring suggested that a 4-point response category structure would be more appropriate than an 11-point response. The rescore was: 0 (no problem); 1–5 (mild problem/does not affect daily life); 6–8 (moderate problem/affects daily life to a certain extent); 9–10 (severe problem/affects all aspects of daily life/life -disturbing). Since few respondents had a score larger than 8 and zero score, four categories were collapsed into two groups for analysis. The independent variables included age, sex, social security, number of vaccine doses, Body Mass Index (BMI), central obesity (≥80 cm for females, and ≥90 cm for males) (34), alcohol drinking, physical activity level, pain severity, sarcopenia, frailty, cognitive status, depression level, anxiety level, loneliness, and social support level. Factors with a value of *p* < 0.1 were entered into the multivariable models to examine the independent predictors. Adjusted OR (aOR) and its 95% confidence interval (CI) were obtained. *p*-value less than 0.05 (two-sided) were considered statistically significant. SPSS version 26.0 (SPSS Inc., Chicago, IL, United States) was used.

## 3. Results

A total of 977 (67.8%) patients completed both baseline and follow-up surveys. Out of these 977 patients, 212 (21.7%) had been infected during the 5th wave of the pandemic; 5 (2.4%) were infected within 4 weeks, 190 (89.6%) were infected in the previous 5–12 weeks and other 17 (8.0%) in the previous 12–24 weeks. Patients infected in the previous 5–24 weeks were included in the analysis.

### 3.1. Post-acute and long COVID-19 symptoms

Among the 207 participants who were infected in the previous 5–24 weeks, the mean age was 70.8 (SD = 5.7) years, and most were female (76.3%). The mean days since COVID-19 infection was 62.5 (18.5) days [median (IQR): 62 (20)] (Table 1). For post-COVID-19 symptoms, 60.9, 56.5, and 30.0% reported fatigue, cognitive difficulty and breathlessness, respectively; 22.2, 32.9, and 19.8% reported one, two and all of the three symptoms, respectively; 46.1% self-reported at least one other new symptom. In total, 81.2% reported at least one post-acute COVID-19 symptom. The average number of symptoms was 1.9 (SD = 1.3).

**Table 1:** Demographic characteristics and COVID-19-related information (*N* = 207).

Variables		
Age (years) (mean ± sd)	70.8 ± 5.7	
≤65	27 (13.0%)	
65–74	129 (62.3%)	
≥75	51 (24.6%)	
Female	158 (76.3%)	
Received COVID-19 vaccination	191 (92.3%)	
Number of doses received		
0	16 (7.7%)	
1	45 (23.6%)	
2	95 (49.7%)	
3	51 (26.7%)	
Type of vaccine received		
Sinovac	117 (61.3%)	
BioNTech	68 (35.6%)	
Mixed	6 (3.1%)	
Days since SARS-CoV-2 infection (mean ± sd)/median (IQR)	62.5 ± 18.5/62 (20)	
*n* (%)		Severity score (mean ± sd)
Post-COVID-19 symptoms (5–24 weeks since infection)	168 (81.2%)	5.4 ± 1.9
Breathlessness	62 (30.0%)	5.3 ± 1.9
Fatigue	126 (60.9%)	5.6 ± 2.0
Cognitive difficulty	117 (56.5%)	5.8 ± 2.0 ^a^
Any of the three post-acute symptoms	155 (74.9%)	5.3 ± 1.9
Other new symptoms	95 (46.1%)^a^	5.8 ± 2.1^b^
Respiratory system symptom (e.g., cough)	29 (14.0%)	
Insomnia/poor sleep quality	29 (14.0%)	
Ear, Nose and Throat (ENT) symptom (e.g., sore throat)	21 (10.1%)	
Musculoskeletal symptom (e.g., bone pain)	9 (4.3%)	
Gastrointestinal symptom (e.g., poor appetite)	7 (3.4%)	
Neurological problem (e.g., dizziness/headache)	6 (2.9%)	
Eye symptoms (e.g., dry eye, blurred eye)	6 (2.9%)	
Heart-related problem	5 (2.4%)	
Ageusia/Anosmia	4 (1.9%)	
Skin problem	3 (1.4%)	
Loss of appetite	2 (1.0%)	
Others	14 (6.8%)	

Post-COVID-19 symptoms included post-acute and long COVID-19 symptoms here. They were symptoms that lasted for 5–24 weeks after infection. Missing: ^a^1, ^b^4. A total of 22 (10.6%) participants had breathlessness at rest (severity score: 4.5 ± 1.8); 13 (6.3%) had breathlessness at dressing (severity score: 3.7 ± 2.1); and 55 (26.6%) had breathlessness when climbing stairs (severity score: 5.4 ± 1.8). For cognitive difficulty, 111 (53.6%) reported short-term memory, 61 (29.5%) reported concentration difficulty, and 44 (21.3%) reported planning difficulty, with 105 (89.7%) reporting that short-term memory was the worst among the three cognitive difficulties.

### 3.2. Predictors of post-acute and long COVID-19 symptoms

Table 2 shows the distribution of the three common symptoms among patients with different characteristics and the univariable analysis results. In the univariable analyses, the female sex, sarcopenia, frailty, depression and anxiety were associated with all three post-COVID symptoms (*p* < 0.1). These variables, together with other variables with p < 0.1, were included in the respective multivariable analyses. Table 3 shows the respective multivariable analyses of the three most common symptoms and the overall severity. Compared to receiving three doses of vaccine, receiving two doses of vaccine was associated with post-acute breathlessness [aOR (95%CI): 2.84 (1.14, 7.05), *p* = 0.025], but no association was found among those with 0 or 1 dose. Higher levels of depression at baseline were associated with post-COVID fatigue [aOR (95%CI): 2.97 (1.06, 8.29), *p* = 0.038 for moderate or above depression], compared to no depression. Females were more likely to have cognitive difficulty [aOR (95%CI): 2.31 (1.12, 4.79), *p* = 0.024]. Additional analysis on predictors of the overall severity score of the three symptoms found a significant association of anxiety at baseline with a higher average severity score. Multivariable analysis on a subgroup of patients with 2 or more chronic conditions (multimorbidity) showed that frailty was significantly associated with fatigue and cognitive difficulty (Supplementary Table S3).

**Table 2:** Risk factors for the presence of post-acute and long symptoms (symptoms last for 5–24 weeks since SARS-CoV-2 infection, *N* = 207).

Variables	Total	Breathlessness	Fatigue	Cognitive function	Overall severity (6–10)^
Age					
≤64	27 (13.0%)	7 (11.3%)	19 (15.1%)	20 (17.1%)	16 (20.0%)
65–74	129 (62.3%)	44 (71.0%)	75 (59.5%)	68 (58.1%)	44 (55.0%)
≥75	51 (24.6%)	11 (17.7%)	32 (25.4%)	29 (24.8%)	20 (25.0%)
Female	158 (76.3%)	54 (87.1%)**	102 (81.0%)*	97 (82.9%)**	67 (83.8%)
Number of COVID-19 vaccination doses received					
0	16 (7.7%)	3 (4.8%)**	9 (7.1%)	10 (8.5%)	5 (6.3%)
1	45 (21.7%)	13 (21.0%)	32 (25.4%)	27 (23.1%)	21 (26.3%)
2	95 (45.9%)	38 (61.3%)	57 (45.2%)	57 (48.7%)	37 (46.3%)
3	51 (24.6%)	8 (12.9%)	28 (22.2%)	23 (19.7%)	17 (21.3%)
Social security^a^	133 (66.2%)	39 (65.0%)	80 (66.1%)	70 (60.9%)*	46 (59.7%)
BMI (kg m^−2^)					
Underweight (BMI <18.5)	9 (4.3%)	1 (1.6%)	7 (5.6%)	7 (6.0%)	4 (5.0%)
Normal (BMI 18.5 – <23.0)	46 (22.2%)	10 (16.1%)	28 (22.2%)	26 (22.2%)	17 (21.3%)
Overweight/Obese (BMI ≥ 23.0)	152 (73.4%)	51 (82.3%)	91 (72.2%)	84 (71.8%)	59 (73.8%)
Central Obesity^b^	147 (71.4%)	51 (82.3%)**	92 (73.0%)	86 (73.5%)	61 (76.3%)
Risky drinker	5 (2.4%)	1 (1.6%)	2 (1.6%)	1 (0.9%)	1 (1.3%)
Physical activity level	106.1 ± 53.2	97.4 ± 35.0*	102.3 ± 50.9	105.0 ± 52.1	101.8 ± 45.4
Pain severity score					
No pain	87 (42.0%)	22 (35.5%)	52 (41.3%)	45 (38.5%)	26 (32.5%)
Mild pain (≤4)	41 (19.8%)	12 (19.4%)	25 (19.8%)	24 (20.5%)	15 (18.8%)
Moderate/Severe pain (>4)	79 (38.2%)	28 (45.2%)	49 (38.9%)	48 (41.0%)	39 (48.8%)
Sarcopenia	42 (20.3%)	17 (27.4%)*	31 (24.6%)*	29 (24.8%)*	24 (30.0%)*
Frailty					
Robust (0)	42 (20.3%)	8 (12.9%)*	18 (14.3%)**	16 (13.7%)**	14 (17.5%)
Pre-frail (1–2)	112 (54.1%)	32 (51.6%)	68 (54.0%)	63 (53.8%)	38 (47.5%)
Frail (3–5)	53 (25.6%)	22 (35.5%)	40 (31.7%)	38 (32.5%)	28 (35.0%)
Cognitive function (MOCA total score)	24.7 ± 3.8	25.2 ± 4.0	24.5 ± 3.9	24.7 ± 3.7	24.3 ± 3.9
Depression					
Normal (0–4)	82 (39.6%)	17 (27.4%)*	37 (29.4%)**	34 (29.1%)**	18 (22.5%)**
Mild (5–9)	75 (36.2%)	28 (45.2%)	50 (39.7%)	46 (39.3%)	30 (37.5%)
Moderate/Moderately severe/Severe (10–27)	50 (24.2%)	17 (27.4%)	39 (31.0%)	37 (31.6%)	32 (40.0%)
Anxiety					
Very mild (0–4)	110 (53.1%)	26 (41.9%)**	59 (46.8%)**	55 (47.0%)**	29 (36.3%)**
Mild (5–9)	57 (27.5%)	25 (40.3%)	37 (29.4%)	32 (27.4%)	25 (31.3%)
Moderate/Severe (10–21)	40 (19.3%)	11 (17.7%)	30 (23.8%)	30 (25.6%)	26 (32.5%)
Social Support (MSPSS total score)	53.2 ± 19.7	52.2 ± 20.8	52.4 ± 19.5	52.3 ± 19.7	51.4 ± 19.3
Loneliness					
Not lonely (0–1)	38 (18.4%)	12 (19.4%)	22 (17.5%)	19 (16.2%)	12 (15.0%)*
Moderately lonely (2–4)	85 (41.1%)	28 (45.2%)	52 (41.3%)	48 (41.0%)	29 (36.3%)
Severely lonely (5–6)	84 (40.6%)	22 (35.5%)	52 (41.3%)	50 (42.7%)	39 (48.8%)

*T*-tests and Chi-square tests were conducted for continuous and categorical variables, respectively. ^*n* = 155; * *p*-value < 0.1, ** *p*-value < 0.05; Missing: ^a^6, ^b^1.

**Table 3:** Multivariable logistic regression for risk factors of post-acute and long COVID-19 symptoms (symptoms last for 5–24 weeks since SARS-COV-2 infection, *N* = 207).

Variables	Adjusted OR (95% CI)	*p*-value
Breathlessness (outcome)		
Number of COVID-19 vaccination doses received (exposure variable)		
3	Ref	
2	2.84 (1.14, 7.05)	0.025*
1	1.60 (0.55, 4.66)	0.388
0	0.84 (0.18, 3.95)	0.825
Fatigue (outcome)		
Depression (exposure variable)		
Normal	Ref	
Mild	1.98 (0.95, 4.10)	0.067
Moderate/Moderately severe/Severe	2.97 (1.06, 8.29)	0.038*
Cognitive difficulty (outcome)		
Gender (exposure variable)		
Male	Ref	
Female	2.31 (1.12, 4.79)	0.024*
Overall severity of the three symptoms (outcome) [mild (1–5) vs. moderate–severe (6–10), *n* = 155]		
Anxiety (exposure variable)		
Very mild	Ref	
Mild	1.38 (0.59, 3.22)	0.458
Moderate/Severe	3.30 (1.16, 9.40)	0.026*

* *p*-value < 0.05. Factors with a value of *p* < 0.1 as indicated in Table 2 were entered into the multivariable models but not shown in that table if their value of *p* > 0.05 after adjusting with other covariates.

## 4. Discussion

### 4.1. Summary

One in five older primary care patients in this prospective cohort was infected during the 5th wave of COVID-19 in Hong Kong. A total of 80% of patients with comorbid physical and psychosocial conditions suffered from post-COVID symptoms: about 60% had fatigue (predicted by depression), 60% had cognitive difficulties (predicted by the female sex), and 30% had breathlessness (predicted by two doses, but not 0 or 1 dose, in reference to three vaccine doses) in the multivariable regression models. Baseline anxiety predicted overall post-COVID symptom severity. In addition, frailty predicted fatigue and cognitive difficulty among those with multimorbidity after infection.

### 4.2. Strengths and limitations

The strength was that this was a prospective longitudinal study that examined various demographic, behavioural, physical, social and psychological predictors of post-COVID symptoms among old primary care patients with comorbid conditions. Risk factors for the three most common post-COVID-19 symptoms were examined. The study had several limitations. First, it might have a selection bias as participation was voluntary. The study only included those patients with complete data at both time points, though multiple calls were made to reach them. The results may not reflect all older patients in primary care, though we believe the rates of infection and symptoms could be higher as usually those who do not respond are those with more severe conditions (35). Second, the sample size may be insufficient for some subgroups and for identifying some potential risk factors (e.g., very few participants received 0 or 1 vaccine dose, or were underweight). But the sample size should be adequate for most risk factors. Future studies with a larger sample size are important to validate our findings. Third, only the three most common post-COVID-19 symptoms were asked using a validated measure, and other post symptoms were self-reported by open question. We might have under-reported some symptoms, though the rate was similar to the rate (76%) reported in another local study of 106 hospital-admitted patients (36, 37).

### 4.3. Comparison with existing literature

The infection rate found in this cohort was higher than the rate (10%) of the general population in Hong Kong in early May 2022 (38). This further implies older primary care patients with comorbid physical and psychosocial conditions as a vulnerable population for COVID-19 infection. The prevalence of post-COVID-19 symptoms was similar to the above local study (36) and twice the global prevalence in a recent meta-analysis: 43% (95% CI: 39–0.46%) (7). Although we only found 3 doses of vaccines had higher protection on post-COVID-19 symptoms compared to 2 doses, but not 0 or 1 dose, this might be due to fewer participants having 0 or 1 dose in the study. Recent population studies showed that 3 doses (mRNA vaccines or a combination of an mRNA vaccine and an inactivated vaccine) provided the best protection against COVID-19 severity (39–41). A recent retrospective matched cohort study using a UK-based primary care database with 486,149 adults found age, female sex, belonging to an ethnic minority, socioeconomic deprivation, smoking, obesity and a wide range of comorbidities (depression, anxiety, migraine, etc.) were risk factors for long COVID (37). The difference might be due to that we had a different study setting and population, adjusted many other various variables as well (e.g., frailty, sarcopenia, physical activity level), and had a smaller sample size. Further studies would be still needed to take a closer look at these risk factors. A study among 54,960 participants in a nurse cohort (predominantly females) in the United States also found that probable depression, probable anxiety, worry about COVID-19, perceived stress, and loneliness early in the pandemic before SARS-CoV-2 infection were associated with a higher risk for post-COVID-19 conditions [risk ratio (RR) ranged 1.32–1.47] (42). The potential reasons for this association might be that those with psychological distress have more chronic proinflammatory cytokines or low-grade microglia activation (42). Regarding gender differences, a recent review showed that the likelihood of having long COVID syndrome was significantly greater among females (OR = 1.22; 95% CI: 1.13–1.32) in general, including diseases of Ear, Nose, or Throat (ENT), gastrointestinal (GI), psychiatric/mood, neurological, dermatological, and other conditions, while the likelihood of endocrine and renal disorders was significantly greater among males (43). The review suggested that differences in the immune system might explain the sex differences. More rapid and robust immune responses with stronger IgG antibody production were seen in the early stages of SARS-COV-2 infection in women, protecting them from infection and severity. However, this might also result in prolonged autoimmune-related disease (43–45). In addition, it is also likely that women are generally more attentive to their body and related distress (46). Overall, there are still many unknowns regarding why depression and sex may predict post-COVID-19 symptoms.

### 4.4. Implications for research and/or practice

First, comparing to two doses, having 3 doses of vaccination was at a lower risk of post-COVID-19 symptoms. However, we did not see a difference of 3 doses comparing to 0 or 1 dose. This may need a further close look of the protective effect of vaccination among this population. Second, to identify at-risk populations with post-COVID-19 symptoms in primary care among older adults with comorbid physical and psychosocial conditions, attention should be paid to those with pre-existing depressive and anxiety symptoms. Future studies are needed to understand the potential mechanisms of these associations for effective intervention design. Studies with evidence-based interventions such as physical exercise, for reducing depressive and anxiety symptoms (modifiable risk factors) may be studied and incorporated into primary healthcare to prevent future outbreaks. In addition, it might need more attention to the sex differences in post-COVID-19 symptoms, its relevant mechanisms, and subsequent interventions. Finally, special attention should be paid to post-COVID symptom severity among frail older adults.

In conclusion, depression, the female sex, frailty and fewer vaccine doses predicted post-COVID-19 symptoms. Future studies should be conducted to explore the potential mechanisms. Promoting vaccination and providing intervention to those at high-risk for post-COVID symptoms are warranted.

## Data availability statement

The raw data supporting the conclusions of this article will be made available by the authors, without undue reservation.

## Ethics statement

The studies involving human participants were reviewed and approved by Joint Chinese University of Hong Kong – New Territories East Cluster Clinical Research Ethics Committee (Reference Number: CREC-2019.329). The patients/participants provided their verbal and written informed consent to participate in this study.

## Author contributions

DZ drafted the manuscript, advised the data analysis and result interpretation, and supported the study implementation. SW conceived the study, obtained the funding, designed and supervised the study, and revised the manuscript. VC contributed to study design, result interpretation, and manuscript revision. DC cleaned, managed, and analyzed the data, and provided the results interpretation and manuscript revision. RL cleaned, managed, and analyzed the data. RS, SM, ZX, WZ, and KT revised the manuscript. All authors contributed to the article and approved the submitted version.

## Funding

This work was supported by the Hong Kong Jockey Club Charities Trust. The funder has no role in study design, collection, management, analysis, and interpretation of data; writing of the report; and the decision to submit the report for publication.

## Conflict of interest

The authors declare that the research was conducted in the absence of any commercial or financial relationships that could be construed as a potential conflict of interest.

## Publisher’s note

All claims expressed in this article are solely those of the authors and do not necessarily represent those of their affiliated organizations, or those of the publisher, the editors and the reviewers. Any product that may be evaluated in this article, or claim that may be made by its manufacturer, is not guaranteed or endorsed by the publisher.
