# Supplementary material for: Determinants of post-COVID-19 symptoms among adults aged 55 or above with chronic conditions in primary care: data from a prospective cohort in Hong Kong
Source: Front Public Health. 2023 May 5;11:1138147. doi: 10.3389/fpubh.2023.1138147 (PMC10196359; doi:10.3389/fpubh.2023.1138147)
Supplement: Supplementary file 1 [file Data_Sheet_1.pdf]

Supplementary Material

Determinants of post-COVID-19 symptoms among adults aged 55 or above with chronic conditions in primary care: Data from a prospective cohort in Hong Kong

Dexing Zhang, Vincent Chi-Ho Chung, Dicken Cheong-Chun Chan, Zijun Xu, Weiju Zhou, King Wa Tam, Rym Chung-Man Lee, Regina Wing-Shan Sit, Stewart W. Mercer, Samuel Yeung-Shan Wong*

*** Correspondence:** Samuel Yeung-Shan Wong: yeungshanwong@cuhk.edu.hk

Supplementary Table 1. Definition of the health condition in the inclusion criteria

Health condition	Measurement	Inclusion criteria
**Physical condition**		
Hypertension	Blood pressure (measured on the assessment day)	⩾ 140/90
Diabetes mellitus	Hb A1c (checked CMS)	>7
Chronic pain	Self reported	Having pain and last for 3 months within last year
Sarcopenia	Sarcopenia assessment (Self reported)	⩾4
Frailty	FRAIL scale (Self reported)	⩾1
Drug compliance	Self reported	Taking ⩾ 5 drugs or forgot to take drug
Underweight	BMI (measured on the assessment day)	⩽ 18.5
**Mental/social condition**		
Depression	9-item Patient Health Questionnaire (Self reported)	⩾ 5
Anxiety	7-item Generalised Anxiety Disorder (Self reported)	⩾ 5
Mild cognitive impairment	Hong Kong Montreal Cognitive Assessment (measured by the assessor)	7th percentile
High loneliness level	6-item De Jong Gierveld Loneliness Scale (Self reported)	⩾ 3
Moderate or low social support	Multidimensional Scale of Perceived Social Support scale (Self reported)	⩽ 5
Living alone or only living with his/her partner	Self reported	Yes

Supplementary Table 2. Chronic condition list

**43 common chronic conditions in 15 categories**	
1. Metabolic disease	hypertension, lipid disorder, diabetes
2. Cancer	
3. Disease of the cardiovascular system	coronary heart disease, stroke/cerebrovascular disease, peripheral vascular disease
4. Disease of the respiratory system	COPD, bronchiectasis, asthma, chronic pharyngitis /laryngitis
5. Disease of the liver, spleen and gallbladder	gallbladder/spleen disease, viral hepatitis, chronic liver disease
6. Disease of the stomach and intestines	dyspepsia and gastritis, diverticular disease of intestine, chronic enteritis; irritable bowel syndrome; constipation
7. Disease of the musculoskeletal and connective tissue	chronic pain needing medication control, skeletal and connective tissue inflammation (such as arthritis, gout)
8. Disease of the genitourinary system	chronic kidney disease (nephritis), prostatitis, benign prostatic hyperplasia
9. Disease of the ear, nose and throat (ENT)	chronic rhinitis, deafness/tinnitus
10. Disease of the visual system	glaucoma/cataracts, blindness/amblyopia, diabetic eyes, retinal detachment
11. Disease of the skin	eczema, psoriasis
12. Disease of the blood	anemia
13. Disease of the nervous system	multiple sclerosis, migraine, epilepsy, Parkinson’s disease
14. Mental disorders	schizophrenia/bipolar disorder, depression, anxiety & other stress related disorders, dementia
15. Others	

Supplementary Table 3. Multivariable logistic regression for risk factors of post-acute and long COVID-19 symptoms (symptoms last for 5-24 weeks since SARS-COV-2 infection) among people with multimorbidity (n=163)

Variables	Adjusted OR (95% CI)	p-value
**Breathlessness**		
Number of COVID-19 vaccination doses received		
3	Ref	
2	4.09 (1.37, 12.24)	**0.012** *
1	2.22 (0.64, 7.68)	0.209
0	2.01 (0.38, 10.56)	0.409
**Fatigue**		
Frailty		
Robust	Ref	
Pre-frail	1.66 (0.67, 4.10)	0.270
Frail	3.21 (1.03, 10.05)	**0.045***
**Cognitive difficulty**		
Gender		
Male	Ref	
Female	2.35 (1.09, 5.08)	**0.030***
Frailty		
Robust	Ref	
Pre-frail	2.33 (0.89, 6.15)	0.086
Frail	5.27 (1.55, 17.92)	**0.008** *

* p-value <0.05
